# ILF-neurofeedback in clinical practice: examining symptom change and performance metrics across diagnostic groups

**DOI:** 10.3389/fnhum.2025.1601187

**Published:** 2025-07-30

**Authors:** Thomas Theis, Ute Bolduan, Sigrid Seuß, Johannes Spallek, Bernhard Wandernoth, René Mayer-Pelinski

**Affiliations:** ^1^REHA Point, Group of Occupational Therapy Clinics in Kassel, Kassel, Germany; ^2^BEE Medic GmbH, Singen, Germany; ^3^Practice for Psychotherapy, Ottendorf-Okrilla, Germany

**Keywords:** neurofeedback, EEG-biofeedback, ILF, infra-low-frequency, continuous performance task, symptom tracking, mental disorders

## Abstract

**Introduction:**

Neurofeedback (NF), particularly Infra-Low Frequency (ILF) Neurofeedback, is an emerging method of neuromodulation aimed at enhancing the brain’s self-regulation. It is a potentially powerful tool to complement the clinician’s toolbox, supporting the treatment of symptoms stemming from arousal regulation deficiencies. Despite the broad use and applicability of the arousal regulation model, there is a gap between its practical use and academic research. This study examines the effectiveness of ILF Neurofeedback across different diagnostic groups and explores whether subjective symptom changes correlate with objective performance measures.

**Methods:**

Between 2015 and 2024, a study of 256 patients in an occupational therapy practice focused on comparing the influence of ILF Neurofeedback on different symptomatic groups. The groups were divided according to the ICD-10 F-codes for “F3—Mood Disorders” (MO), “F4—Neurotic, Stress-Related, and Somatoform Disorders” (NS), “F8—Developmental Disorders” (PD), and ‘F9—Childhood/Adolescent Behavioral Disorders’ (BE). Symptom tracking and the Continuous Performance Test (CPT) for assessing errors and reaction times were used to monitor progress before and after neurofeedback therapy.

**Results:**

Discriminant analysis showed significant symptom profile differences across diagnostic groups with an accuracy of 79%. A linear mixed model revealed consistent symptom reduction over Neurofeedback sessions, with a faster decline in early sessions. ILF Neurofeedback improved response times, reduced errors, enhanced discriminative ability, and increased caution, with no group differences. Correlation analysis showed that symptom tracking correlated with reduced commission errors and improved d-prime in the MO group, while in NS, it was linked to d-prime increase. In PD, symptom tracking correlated with correct responses and fewer omission errors; no significant correlations were found in BE.

**Discussion:**

This study confirms that ILF Neurofeedback is equally effective across four diagnostic groups regarding self-report and performance. Symptoms significantly decreased during NF, with the fastest decline in the first 10 sessions. Performance improvements were seen in Continuous Performance Test measures, but symptom decline only correlated with performance in some groups. This suggests that subjective ratings and performance may be independent or depend on the diagnostic group. Further research with a control group is needed to explore ILF’s effects.

## 1 Introduction

Given the global rise in psychiatric conditions (Kohring et al., 2024; UNRIC, 2022), the demand for treatment is growing. Two predominant paradigms in mental health treatment are psychotherapy and pharmacotherapy. While psychotherapy is based on learning processes, pharmacotherapy targets presumed chemical imbalances. Yet, even in countries with well-developed psychotherapy systems, waiting times can stretch over months (Funke-Kaiser, 2022). Pharmacotherapy, although widely used, often comes with side effects that reduce the quality of life (Schein et al., 2023), and its beneficial effects may disappear when medication is discontinued. Additionally, some patients do not respond (Childress and Sallee, 2014) or fail to adhere to medication (Adler and Nierenberg, 2010). In critical cases, such as epilepsy, where patients rely on medication (Kwan and Brodie, 2006), limited alternatives may lead to invasive last-resort options like neurosurgery. Psychotherapy itself faces challenges such as long waiting times, high dropout rates (Leichsenring et al., 2019), and sometimes non-response to the treatment (Semmlinger et al., 2024; Vittengl et al., 2016). Thus, while both dominant paradigms remain the cornerstone of mental health treatment, their limitations in scalability, accessibility, and sometimes effectiveness highlight the potential value of complementary approaches such as neurofeedback. Neurofeedback offers a complementary approach grounded in the idea that dysregulations in brain activity contribute to the development of mental disorders (Broyd et al., 2009; Takahashi, 2013).

Neurofeedback is a time-limited, learning-based intervention that enables individuals to regulate their brain activity, often resulting in lasting improvements (Van Doren et al., 2019). It has shown promise in treating depression (Chen and Lin, 2020), ADHD (Riesco-Matías et al., 2021), autism spectrum disorder (Saleem and Habib, 2024), anxiety, and PTSD (van der Kolk et al., 2016), yet remains underrepresented in epilepsy despite given evidence (Tan et al., 2009). There are likely about 40 times more scientific publications on psychotherapy than on neurofeedback, but evidence has been growing over the past years.

Neurofeedback has been a growing research field since its birth. It is a method that uses methods such as EEG, fMRI, or fNIRS for measuring parameters of brain activity. These parameters are made visible to the central nervous system of the client via auditory, visual, or tactile feedback. The perception of the generated feedback signals by the various sensory organs is understood to act like a “mirror for the brain,” which then can react to its own previously unobserved activity. One of the main hypotheses is that this interaction results in enhancing the self-regulation capacity of the brain and thereby optimizing brain function.

However, up to now, the working mechanisms of neurofeedback are not fully understood, partly because the mechanisms of self-regulation are not fully understood. One suggestion is that self-regulation is driven by the ability of the brain to switch more flexibly between the different neuronal networks (Dobrushina et al., 2020), while other conceptualizations focus more on arousal optimization (Ros et al., 2014).

Since the early work on neurofeedback in the 1970s, various methods have been developed, which differ in the data extraction from the brain signal and the implementation of the feedback. In the method often referred to as the frequency band method, the feedback signal is a reflection of the increase or decrease of specific frequency ranges in the EEG spectrum (1–40 Hz). In neurofeedback based on slow cortical potentials (SCP), brain activity in the low-frequency range below 0.1 Hz is used for feedback and training purposes. In SCP neurofeedback, as developed in Europe, the client is asked to actively control the feedback signal and therewith learn to control mental processes (Aggensteiner et al., 2019; Birbaumer et al., 1981).

A different way to utilize the SCP signal, called infralow frequency neurofeedback (ILF) was developed in the Anglo-American region. This method utilizes a continuous stream of feedback from frequencies below 0.1 Hz. In addition, the frequency bands from 1 to 40 Hz are used for feedback to discourage any sudden larger excursions. The specific range of the infralow feedback signal is determined by the response of the client during and after the session (Othmer et al., 2013). For an adaptation of this method and its clinical use (see Kirk, 2015; Leong et al., 2018; Smith et al., 2014). It is important to note that rigorous clinical observation by neurofeedback pioneers gave birth to ILF. However, there is a gap between the wide applicability and its observed effectiveness compared to the academic publications of ILF. One effort to close this gap was the recently published special issue in Frontiers in Human Neuroscience on the “Endogenous Neuromodulation in the Infra-Low Frequency Regime” (Othmer, 2022) covering the applications of ILF to a broad spectrum of indications such as frontal brain lesion and headaches, but also autism spectrum disorder and trauma.

The broad applicability of ILF results from its orientation to see symptoms as indicators of over—or under-arousal and stability, which is the so-called arousal model (Fisher, 2014; Legarda et al., 2011).

In addition to continuous behavioral observation of the client during the session, symptom tracking is primarily used as an initial assessment and for monitoring progress. Similar to symptoms assessed in psychopathology (Stieglitz et al., 2017), this involves a set of questions about symptoms specifically relevant to neurofeedback as indicators for assessing arousal regulation. The symptom tracking questionnaire, originally developed by Sue Othmer, collects both psychological and somatic symptoms that have proven to be relevant for assessing the brain’s level of arousal regulation. The intention behind the development of this questionnaire was not to identify diagnostic categories but to support the neurofeedback process and reflect on its effectiveness. This can aid in adjusting training parameters for further sessions.

In addition to self-reports of symptoms in symptom tracking, it has also become an established practice to collect performance measures for assessment and progress monitoring. They are used to investigate arousal regulation and cerebral stability by evaluating different indicators, such as commission and omission errors, but also the reaction time of the client.

A CPT is often used for the assessment of ADHD (Varela et al., 2024), and less frequently also for affective disorders (Koetsier et al., 2002). However, CPTs are now cited as the most frequently used measure of attention in both practice and research (Riccio et al., 2002).

To our knowledge, there are no investigations on how and if those two types of measures, subjective report and objective performance, relate when ILF is applied in a naturalistic setting. Therefore, symptom tracking and continuous performance test data were collected for a large group of clients.

In psychological care, diagnosis according to diagnostic systems like ICD (Dilling and Dittmann, 1990) is relevant for treatment selection and reimbursement. This raises the question of whether the more symptom-oriented approach based on the arousal model yields comparable results across diagnostic classifications. The present work aims to answer the following research questions:

1. Is ILF Neurofeedback equally effective across diagnostic groups?
2. Are there differences in the subjective rating measured by the symptoms tracking questionnaire across different diagnostic groups?
3. Are there differences in the objective measures/performance across diagnostic groups?
4. Does the magnitude of symptom change correlate with the improvement of performance?

## 2 Materials and methods

### 2.1 Patient group

Symptom tracking, continuous performance test data and diagnostic ICD-10 F-code categories were collected in an occupational therapist practice between 2015 and 2024. A requirement for treatment was that the patient was already stabilized, if medication was taken, with no further changes planned or expected. Informed consent is obtained for both neurofeedback training and data handling before proceeding.

The intake process consists of an interview recording the personal and illness history, the symptom tracking along with patterns of dysregulation, and the CPT (QIK-Test) assessment. The focus is on identifying arousal indicators, instabilities (paroxysmal symptoms), disinhibition, localized dysfunctions, and learned fears and habits. The focus specifically was on cases with F-codes falling under the primary categories of “F3—Mood [Affective] Disorders” (MO), “F4—Neurotic, Stress-Related, and Somatoform Disorders” (NS), “F8—Disorders of psychological development” (PD), and “F9—Behavioral and emotional disorders with onset usually occurring in childhood and adolescence” (BE) for subsequent analysis. The ICD-10 F-codes were provided by the referring clinician, with neurofeedback being administered by occupational therapists in the German healthcare system upon referral from a general physician, psychiatrist, or psychotherapist. The overall sample comprised of *N* = 256. A total of 36 out of 256 cases could not be analyzed due to missing data points, corresponding to approximately 14.1% incomplete cases. The attrition rate was zero, meaning no patient discontinued the treatment. An overview of the clients is given in Table 1, which shows the number of clients per group, the mean age, the gender distribution, and the mean number of neurofeedback sessions.

**TABLE 1:** Sample characteristics.

F-category	N	age	(SD)	Gender% male	Sessions	(SD)
MO	28	44.70	(14.93)	35	30.19	(21.55)
NS	44	40.13	(17.57)	41	30.25	(26.01)
PD	45	10.90	(9.40)	57	35.11	(29.68)
BE	137	17.46	(13.63)	69	25.36	(18.37)

*N*, sample size; sessions, neurofeedback sessions.

### 2.2 ILF neurofeedback

In this data collection, ILF Neurofeedback was applied using a NeuroAmp (Corscience, Germany) amplifier and the Cygnet (BEE Medic GmbH, Germany) Neurofeedback software, utilizing full bandwidth (DC to 130 Hz), 32-bit resolution, a sampling rate of 500 sps, and an integrated impedance meter (impedance range 0–140 kOhm).

The neurofeedback protocol uses two channels with a common reference electrode at Cz. The inhibit signal was computed by summing both channels. The monitored frequency range, 1–40 Hz, was divided into eight bands for analysis. For each band, a threshold was set so that the signal remained below 95% of the time. The average distance from the threshold modulated feedback elements, such as volume and fog. For example, the specific electrode placement T4–P4 would be implemented as Channel 1 = T4–Cz and Channel 2 = P4–Cz. The inhibit signal was calculated as T4 + P4—2 Cz. The reward signal was derived from the difference between both channels: (T4–Cz)—(P4–Cz) = T4–P4, consisting of the slow cortical potential in the infralow range below 1 Hz. This ILF signal controlled the animation speed, brightness, and color saturation of the feedback given to the patient. Patients select a preferred feedback modality, such as a movie or animation.

Based on all gathered information (symptom tracking, QIK, and interview—each of which is described further below), the initial electrode placement at T3-T4, T4-P4 or both is selected. Depending on localized dysfunctions, prefrontal positions (T3-Fp1, T4-Fp2, or Fp1-Fp2) are then added in the progress of the therapy.

For each client, the electrode position and the optimum training frequency is adjusted according to the protocol guidelines (Othmer, 2012). Before the electrodes are applied, the skin at the electrode site is cleaned with an abrasive paste (Nuprep^®^, Weaver and Company, United States). The Ag/AgCl electrodes are placed using a conductive paste (Ten20^®^; Weaver and Company, United States) to ensure sufficient conductivity. The optimal reward frequency is determined within the first 1–3 sessions, based on patient reports or clinician observations of arousal and underarousal indicators (Wiedemann, 2020).

### 2.3 Symptom tracking

Symptom tracking, commonly used in neurofeedback therapy, involves monitoring relevant symptoms throughout the course of the treatment. The symptom tracking questionnaire includes seven categories, each with 10–12 items (see Supplementary Appendix A). Clients assess up to 25 relevant items (categories: sleep, pain, attention and learning, sensory processing, behavior, emotion, and physical health) related to their symptoms, forming a personalized symptom profile. Each symptom is tracked over time, allowing for continuous monitoring of symptom progression. Symptom tracking was done weekly before each session.

### 2.4 Continuous performance test—QIK

The QIK test (BEE Medic GmbH, Germany) is a continuous performance test that runs for 21 min. It is a go/no-go challenge test. On the handheld device different patterns in an array of nine lights light up and depending on the pattern, the client has to press a button. When all lights flash, the client is not allowed to press a button, whereas when all lights but the center one flash, the client has to press a button as quickly as possible. The results are evaluated using EEG Expert (Versace, 2025). Information about the commission and omission errors, the reaction time, and the standard deviation is gained. Signal detection measures were used to unconfound discrimination ability (d-prime *d’*), the capacity to differentiate targets from non-targets and from response bias, a general tendency to say “yes” or “no” (decision criterion/bias *c*) (Green, 1966; Kurysheva et al., 2022). The QIK test was given 1 week before the first and 1 week after the last neurofeedback session. CPT performance reflects the nervous system’s functional state optimized for task performance, particularly in distinguishing signal from noise—an essential ILF-Neurofeedback outcome. It offers diagnosis-independent insight into self-regulation capacity through the lens of the arousal model and treatment effects.

### 2.5 Data analysis

All data preparation and analysis were conducted using the statistical environment *R 4.2.2* (R Core Team, 2024) using the packages tidyverse (Wickham et al., 2019), TInPosition (Beaton et al., 2014), and lme4 (Bates et al., 2015).

Group differences in response profiles were examined using discriminant correspondence analysis (Beaton et al., 2016; Greenacre, 2017). Longitudinal symptom changes were analyzed with linear mixed-effects models, including log-transformed time and diagnostic group as fixed effects and subject-specific random effects (Snijders and Bosker, 2011). Logarithmic symptom trends were identified through exploratory data analysis. QIK outcomes were modeled using mixed-effects models with normal or Poisson distributions, depending on the variable type (Zuur et al., 2009). Pearson correlations were used to assess relationships between changes in symptoms and performance.

## 3 Results

### 3.1 Symptom tracking

#### 3.1.1 Response profiles

Counts per item for each F-category (for a detailed listing, see Supplementary Appendix B) are utilized to conduct a discriminant correspondence analysis (Beaton et al., 2016). This multivariate approach aims to highlight differences in response profiles and creates a representation in a lower-dimensional space (Greenacre, 2017). It helps to determine if groups are distinguishable in terms of response profiles and identifies similarities or differences between them, along with identifying which items contribute to these distinctions.

The analysis resulted in a highly significant solution (*p* = 0.002, inertia = 0.27, *r*2 = 0.50), with significant eigenvalues for the three axes (lambda1 = 0.184, *p* = 0.001; lambda 2 = 0.0.046, *p* = 0.001; lambda 3 = 0.035, *p* = 0.001). The model’s accuracy in terms of F-category prediction was 79%.

**FIGURE 1:**
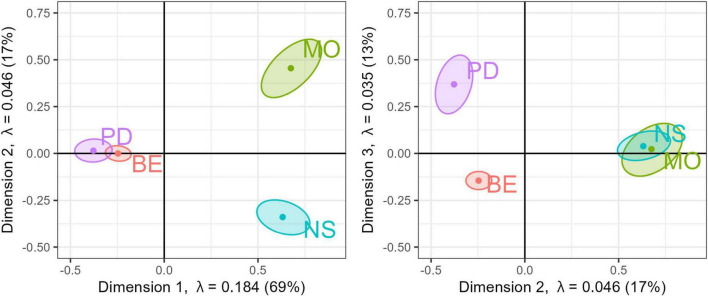
Factor map. Three-dimensional solution from a discriminant correspondence analysis. (Left) factor map dimension 1 vs. dimension 2; (right) factor map dimension 2 vs. 3. with the corresponding eigenvalues and percentage of variance explained. Points show color-coded group-mean F-category with the corresponding confidence intervals.

Figure 1 displays the factor map illustration with group means and confidence intervals from two different perspectives. The center of each ellipsoid represents the mean response profile for each group, while the ellipsoid reflects the confidence interval. Proximity and distance of points represent similarity in group profiles. Non-overlapping confidence intervals indicate that the group response profiles are reliably different. Dimension 1 (x-axis) separates “development” (PD, BE) from “mood, anxiety, stress” (MO, NS), capturing 69% of the variance. Dimension 2 (y-axis) further separates MO from NS and BE from PD, accounting for 17% of the variance. Dimension 3 separates PD from BE with 13% variance. Overall, MO and NS forming one cluster, and PD and BE form another.

Items that wield substantial influence on the first dimension, distinctly setting apart MO and NS from PD and BE, are distractibility, attention, and concentration as opposed to depression, panic attacks, and pain. Similarly, items contributing to the second dimension, primarily distinguishing MO from NS, involve depression and lack of sense of humor versus flashbacks and heart palpitations. In the third dimension, where BE is predominantly separated from PD, noteworthy contributors encompass hyperactivity, lack of alertness vs. reading difficulty, and poor fine motor coordination. See Supplementary Appendix C for a complete table of item contributions, with bootstrap *t*-value by dimension statistics. Largely, the items with the greatest influence on each dimension also reflect the diagnostic symptoms characteristic of the respective disorder class. However, this is not always the case, nor is it necessary to expect, as arousal indicators do not necessarily map onto diagnostic categories. Nevertheless, the analysis shows a tendency for different response profiles to emerge for each diagnostic group.

#### 3.1.2 Longitudinal symptom change of average symptom rating

To examine the temporal progression of symptom intensity, ratings (ranging from 1 to 10, indicating absence to strong presence) across all items of one patient were averaged at each time point, yielding a time series representing the mean symptom strength per patient.

Subsequently, a linear mixed model with a normal error distribution was employed for data analysis. Here’s the corrected version: The symptom ratings were modeled as fixed effects for time (i.e., number for successive neurofeedback sessions) and group (MO, NS, PD, BE), with subject-wise random effects for intercept and slope (Snijders and Bosker, 2011). Exploratory data analysis suggested that the impact of time on symptom ratings was best described by a logarithmic linear trend, i.e., trend line.

The Analysis of Variance indicates a significant main effect of the logarithm of the session on the response variable. Specifically, log (session) accounts for a substantial proportion of variance, *F*(1, 226.34) = 390.03, *p* < 0.001, suggesting a strong relationship between session progression and the outcome. Specifically, average symptom ratings decline significantly to *b* = −1.097, se = 0.093, *t*(230.99) = −11.75, *p* < 0.001, Cohen’s *d* = −1.81 (−2.05, −1.58). This leads to reductions in symptom decline over 5-session intervals (sessions 1–5, 6–10, 11–15, and 16–20) of 24, 14, 9, and 7%, respectively.

**FIGURE 2:**
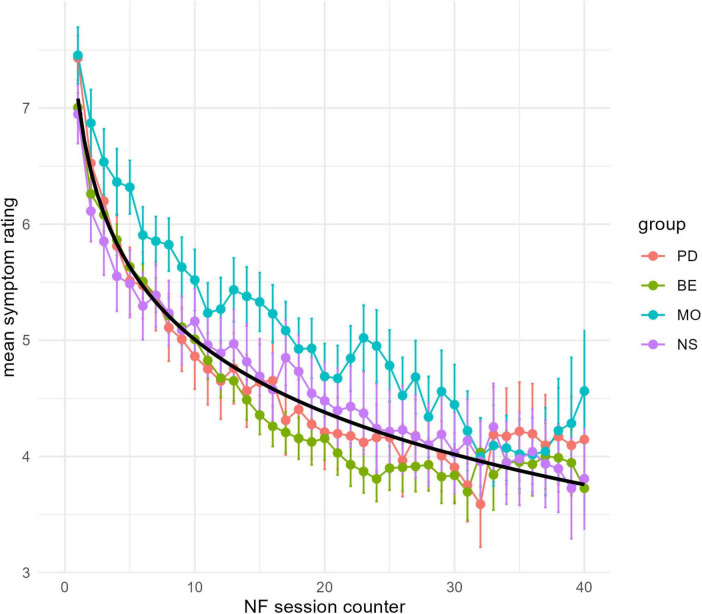
Symptom ratings. X-axis Neurofeedback Session counter; Y-axis mean symptom severity rating by group (MO, NS, PD, BE) color coded and the prediction by the linear mixed model (black line). Error bars show standard deviations.

The main effect of the group factor was not statistically significant, *F*(3, 249.91) = 1.53, *p* = 0.207, indicating no substantial differences across the levels of the group. The interaction between log (session) and group did not reach significance, *F*(3, 227.97) = 2.26, *p* = 0.082, which implies that the effect of average symptom decrease over time is independent of group. Figure 2 shows the model prediction at a population level as well as the average symptom change per group.

### 3.2 Continuous performance test—QIK

The QIK test yielded several analyzed outcome measures, including reaction time (RT), standard deviation (SD), commission errors (Ncommission), omission errors (Nomission), and scores derived from signal detection theory: d-prime and criterion. The inclusion of d-prime and criterion aimed to delve deeper into the potential effects of NF on performance, particularly to discern whether any observed alterations were attributed to changes in sensitivity, response criterion, or a combination of both factors. Table 2 shows the mean outcomes by diagnostic groups and time points (before the first session and after the final NF session).

**TABLE 2:** QIK measures.

Variable	MD		NS		PD		BD	
	Before	After	Before	After	Before	After	Before	After
RT [ms]	399.96	382.14	409.70	397.90	487.00	489.31	454.23	438.17
SD	68.35	62.60	69.34	63.70	129.84	114.93	109.38	99.55
Omission	4.35	4.78	7.04	4.06	31.91	34.48	33.83	26.20
Commission	9.10	5.17	6.59	4.36	41.88	23.77	34.14	24.07
Correct	264.14	259.39	261.97	265.06	230.71	232.13	230.75	241.11
D-prime	4.55	4.85	4.65	4.85	2.75	3.28	3.03	3.58
c	−0.18	−0.09	−0.08	−0.02	−0.03	0.11	−0.02	0.01

Errors of omission and commission, and number of correct responses, signal detection measures: d-prime, criterion c by F-category before and after ILF-NF.

For each QIK measure, a mixed model was calculated with *time* (before, after NF) * *group* (MO, NS, PD, BE) as fixed effects and subject-wise random slopes and intercepts. The two error count measures (omission and commission) were modeled using a Poisson distribution, else a normal distribution was used (Zuur et al., 2009).

#### 3.2.1 Response time

The results of the analysis revealed a significant main effect of *time* [*F*(1, 251) = 5.31, *p* = 0.022], indicating a reduction in the response time after Neurofeedback [β = −16.058, *t*(251) = −2.97, Cohen’s *d* = 0.1457]. Additionally, there was a significant main effect of the group [*F*(3, 321.36) = 8.37, *p* < 0.001], suggesting that groups differed in average response time. In this and for the following measures, we will not elaborate on this *group’s* main effect, as it is not important for our hypotheses. The interaction effect between *time* and *group* was not significant [*F*(3, 251) = 1.03, *p* = 0.381], indicating that the changes in response time over time were consistent across the groups.

#### 3.2.2 Standard deviation

The analysis results revealed significant main effects of both *time* and *group* on the dependent variable standard deviation. The effect of *time* [*F*(1, 251) = 24.12, *p* < 0.001] indicates a significant change in the outcome over time. Specifically, there was a significant reduction of SD after NF [β = −9.826, *t*(251) = −4.65, Cohen’s *d* = 0.3304]. Additionally, the main effect of the group [*F*(3, 332.75) = 37.59, *p* < 0.001] demonstrates significant differences in the SD across the four groups. The interaction effect between *time* and *group* [*F*(3, 251) = 1.29, *p* = 0.278] was not significant, suggesting that the changes over time were consistent across the groups.

#### 3.2.3 D-prime

The analysis results revealed significant main effects of both *time* and *group*. The effect of time [*F*(1, 251) = 25.45, *p* < 0.001] indicates a significant increase in d-prime after NF [β = 0.54939, *t*(251) = 6.108, *p* < 0.001; Cohen’s *d* = 0.3380]. Additionally, a main effect of the group [*F*(3, 345.51) = 27.85, *p* < 0.001] demonstrates significant differences in d-prime across the four groups at baseline. However, the interaction effect between *time* and *group* [*F*(3, 251) = 1.48, *p* = 0.22] was not significant, suggesting that the changes over time were consistent across the groups.

#### 3.2.4 Criterion

The results of the analysis revealed a significant main effect of *time* [*F*(1, 254) = 2.73, *p* = 0.007], indicating a positive shift in the criterium over time [β = 0.06757, *t*(254) = 2.73, *p* = 0.007], which reflects a slight shift from liberal (“signal is present”—tendency) to conservative, resulting in a reduction in the false alarm rate. Additionally, there was a significant main effect of *group* [*F*(3, 251) = 29.26, *p* < 2.94 × 10], suggesting that groups differed significantly in the average baseline criterion used.

#### 3.2.5 Omission errors

The analysis resulted in significant main effects for both *time* [χ^2^(1) = 92.064, *p* < 0.001] and *group* [χ^2^(3) = 87.953, *p* < 0.001], reflecting substantial differences in error rate across time and between groups. However, there was no significant interaction effect between time and *group* [χ^2^(3) = 2.2758, *p* = 0.5172], suggesting the effect of time did not depend on groups. The coefficient for *time* is *b* = −0.6262, *p* > 0.001, indicating a significant decrease in commission errors, with the incidence rate decreasing by approximately 46.6%. Importantly, the interaction term by group is not significant, so the effect of *time* is similar across groups.

#### 3.2.6 Commission errors

The analysis reveals that both *time* [χ^2^(1) = 110.394, *p* < 0.001] and *group* [χ^2^(3) = 115.030, *p* < 0.001] have highly significant effects on commission errors. Specifically, the coefficient for *time* is *b* = −0.71039, *p* = 1.94 × 10−15, indicating a significant decrease in commission errors, with the incidence rate decreasing by approximately *49.1%*. Importantly, the interaction term by group is not significant (χ^2^ = 1.348, *p* = 0.7178), suggesting that the time effect is not different between groups.

To sum up across all measures, there was a consistent pattern of improvement after ILF, particularly faster response times, reduced errors, greater discriminative ability, and the tendency to be more cautious after NF. The lack of significant interaction effects between time and group across most measures suggests that the changes over time were consistent across all groups.

### 3.3 Relationship between performance and symptom tracking

To examine the potential correlation between changes in ratings (symptom tracking) and performance (QIK Test), the slope of the symptom tracking and the slope across all QIK performance measures for each participant were extracted.

A correlation analysis was conducted with all scaled change measures (see Table 3).

**TABLE 3:** Correlations.

Measure	MO	NS	PD	BE
RT	0.28	−0.19	−0.23	−0.06
SD	0.35	0.05	0.11	0.10
Commission	0.41*	0.20	0.07	0.12
Omission	0.20	0.22	−0.38*	0.12
Correct	−0.23	−0.25	0.35*	−0.15
D-prime	−0.53*	−0.31*	0.13	−0.17
c	0.03	−0.07	−0.38*	0.01

Correlations between changes in QIK measures and changes in symptom tracking for each F-category by Errors of omission and commission, number of correct responses, d-prime, criterion c. *Asteric marks statistical significance.

Within the MO group, a significant correlation was found between the symptom tracking slope and commission errors decline [*r* = 0.412, *p* = 0.030, *t*(26) = 2.30], indicating that a stronger reduction in commission errors significantly corresponds to a decline in symptom ratings. Additionally, a significant correlation effect was observed for symptom tracking slope and d-prime increase [*r* = −0.527, *p* = 0.004, *t*(26) = −3.16], revealing that a larger positive change in d-prime corresponds with a greater reduction in symptom ratings.

For the NS group, a significant correlation effect was observed for symptom tracking slope and d-prime increase [*r* = −0.309, *p* = 0.04, *t*(42) = −2.11], revealing that an increase in d-prime corresponds with a greater reduction in symptom ratings.

A significant correlation between the number of correct responses and symptom tracking slope [*r* = 0.35, *p* = 0.02, *t*(43) = 2.44] and a negative correlation between the omission errors and symptom tracking slope [*r* = −0.38, *p* = 0.01, *t*(43) = −2.66] as well as a negative correlation [*r* = −0.38, *p* = 0.01, *t*(43) = −2.66] between symptom reduction and criterion shift was observed in the PD group.

Within the BE group, none of the performance measures correlated with symptom tracking.

## 4 Discussion

Publications on ILF Neurofeedback frequently present case studies or case series with small sample sizes, highlighting the versatile applicability and feasibility of this approach. However, case reports limit the ability to draw strong conclusions. Randomized controlled trials (RCTs) help ensure that the observed effects are due to the intervention itself rather than group-specific differences. However, RCTs should be complemented by observational studies in real-world settings. This observational study presents data from a large cohort of clients in a naturalistic environment, including their diagnostic categories. We addressed the research question of whether Neurofeedback can be beneficial for reported symptoms and behavioral measures across different diagnostic groups. Despite each diagnostic group having a distinct symptom profile with a unique set of complaints, the results showed a decrease in average symptom ratings across all four groups (BE, PD, MO, NS) following NF treatment. This means that the statistical analysis is designed to show that the different groups represent statistically different sets of symptoms.

To our knowledge, this is the first study to examine symptom development over time with high resolution, using measurements at each neurofeedback session. Case studies often used symptom tracking as the main endpoint but were limited to single cases (Sasu, 2022; Spreyermann, 2022). Here, we present average symptom changes across hundreds of cases. Results show that improvement follows a logarithmic curve, with the strongest effects at the beginning and slowing over time. While using a non-validated symptom questionnaire is a limitation, it aligns with ILF Neurofeedback in practice, where symptom tracking is a tailored instrument designed to capture categories relevant to the arousal model. Symptom tracking prioritized these changes over diagnostic categories, limiting conclusions on diagnostic status. Correspondence analysis suggests a link to underlying disorders, but further validation is needed. Future research should incorporate disorder-specific instruments, clinically validated symptom lists like the SCL-90 (Derogatis, 1983), and emotion-specific instruments like the STAI (Spielberger et al., 1970) to clarify diagnostic significance.

However, Fleischman (2022) used a different tool, similar to goal attainment scaling and found effects over time—both in curvature (rapid initial improvement) and change amplitude—that are consistent with our findings.

NF effectiveness was also reflected in CPT measures, with faster, more consistent response times and fewer errors. However, the link between symptom decline and performance gains varied by diagnostic group and specific performance measure. In MO and NS, symptom decline was associated with improved sensitivity, whereas in PD, the error rate decreased with symptom improvement. No correlation was found in BE.

Since these data were collected in a naturalistic setting, there are potential alternative explanations for the symptom decline that may not be directly related to the neurofeedback treatment. First, symptom reduction could result from spontaneous recovery or positive events during treatment. Second, ILF Neurofeedback is sometimes used alone or in conjunction with other therapies, meaning factors such as life events, additional treatments, or patient expectations may also contribute to symptom improvement. Another potential confounder could be learning effects; however, the QIK is modeled after the TOVA, for which no learning effects have been reported (Rotem et al., 2019). The lack of a control group in this naturalistic study makes it difficult to rule out alternative influences. However, despite potential confounding factors like concurrent therapies (pharmacotherapy, psychotherapy) or life events, we argue that the observed symptom reduction is unlikely to be solely due to these factors for the following reasons. Individuals undergoing neurofeedback are often from a waiting list and may or may not receive other treatments beforehand. In many cases, Neurofeedback is used as an alternative or supplementary therapy method when other therapies fail to work or are not long-lasting, such as medication. However, symptom severity remains high at the start of ILF Neurofeedback (as can be seen in the symptom tracking profiles), and spontaneous improvement is rare. Therefore, the reported symptom reduction and performance gain are likely to be at least partly due to the Neurofeedback treatment, regardless of the diagnostic group.

The overall effect of psychotherapy is composed of general (common) factors, specific therapeutic elements, and placebo-related mechanisms (Enck and Zipfel, 2019) that are estimated to account for 15–40% of the total therapeutic effect. The specific effect size of ILF is beyond the scope of this retrospective study; however, existing RCTs (Annaheim et al., 2022; Bakhtafrooz et al., 2025; Carlson et al., 2025; Winkeler et al., 2022) report effects of ILF beyond those of control conditions. However, it is plausible that expectancy effects contributed to the outcomes as a form of placebo effect. ILF-Neurofeedback likely influenced brain activity, leading to improved performance and subjective ratings. But what underlying processes might explain this effect? Oscillatory dynamics in the traditional EEG frequency bands have been observed to be linked to fluctuations in sustained attention, including lapses (Tran et al., 2020). Additionally, spontaneous variations in Default Mode Network signal levels have been shown to predict performance in fMRI studies (Kucyi et al., 2016). Neurofeedback has been demonstrated to influence event-related processes, such as normalizing the P3 component (Askovic et al., 2020). However, the mechanisms by which ILF-Neurofeedback enhances performance or alleviates symptoms remain unclear. In a randomized controlled study by Dobrushina et al. (2020), the effect of one session of ILF-Neurofeedback was investigated. Resting-state fMRI data of 52 participants receiving either real or sham sessions were compared. Results showed increased connectivity in salience, language, and visual networks after Neurofeedback, suggesting enhanced sensory integration. A recent fMRI RCT by de Matos et al. (2025) found that even a single ILF session leads to distinct activation patterns compared to sham. Further research is necessary to determine the extent and significance of ILF’s effects.

The group-specific correlation between changes in subjective ratings and performance, or the lack of correlation in certain groups or measures, is quite surprising. One explanation is that symptoms and performance are independent dimensions of health and not directly related. Another possibility is that the correlation may exist in more specific diagnostic categories not included in this study, but becomes diluted in larger, more diverse diagnostic groups. Previous research often focused on the question of whether specific diagnostic conditions can benefit from ILF neurofeedback (Grin-Yatsenko et al., 2018; Schneider et al., 2021). In this naturalistic study, the effectiveness in terms of subjective ratings and performance is shown for different diagnostic groups. These results are consistent with the predictions of the arousal regulation model, in which the state of arousal, rather than the diagnostic group, determines the specific neurofeedback protocol. Although it is a plausible assumption that subjective symptom loss goes along with a performance gain, those two domains seem not to be correlated in a simple overall manner. Although this seems plausible, it is only partially supported by evidence, for example, in the ADHD literature (Edwards et al., 2007). Performance and subjective ratings could be orthogonal endpoints, or they could be conditionally dependent on the diagnosis.

A limitation of this study is the lack of an active control group, as longitudinal data from a waitlist or symptom tracking in a control group is uncommon in naturalistic settings. Furthermore, as the data were collected from routine therapy practice, the specific effect of NF over other ongoing processes cannot be distinguished. However, as mentioned before, it is unlikely that the symptoms would have decreased over time on itself. Moreover, the referral to NF as an alternative treatment often originates because traditional treatments before did not yield the desired result. Thus, it is plausible to attribute the effects to the NF. Another limitation is that the sample size, age, and gender were unevenly distributed over the different diagnostic groups. Specifically, the relatively lower overall baseline performance in the PD and BE groups in comparison to the other groups might be caused by the younger age in this group. More importantly, group differences are not reflected in symptom decreases or overall performance gain.

As with other complex interventions, dismantling is important to identify which components drive therapeutic change. However, this should follow only after robust baseline effects have been established—otherwise, breaking down the protocol risks missing the synergistic impact of its integrated elements. Specifically, future analyses should include the addition of follow-up placements, such as T4-Fp2, as time-varying covariates to show the specific effect at the single-item level. Greater intervention complexity increases variability in therapeutic skill, necessitating the inclusion of therapist-level covariates like years of experience in future analyses.

Another shortcoming of this study is that the diagnostic groups by themselves are defined broadly; specifically, we have only considered the first number after the F-code because a finer-grained resolution would take too many years or a multicenter approach. On the other hand, ILF Neurofeedback works with the arousal model, which is symptom-driven rather than syndrome or diagnosis-driven. In line with this approach, the current study demonstrates that there is no differential effect of ILF-NF in these broad groups. The results demonstrate the actual course of applied NF treatment in a realistic setting.

## 5 Summary

This study, conducted with a large cohort in a naturalistic setting, confirms that ILF NF is equally effective in terms of self-reporting and performance over four diagnostic groups.

Despite the differing symptom profiles across the four diagnostic groups, there was a notable decline in average symptoms over the course of NF. The session-wise symptom tracking shows that the rate of symptom loss seems to be accelerated most at the first 10 sessions and then levels off. Moreover, there was an improvement in performance over various measures of the CPT. However, only in some diagnostic groups was the symptom decline associated with the performance improvement. This suggests that subjective ratings and performance may be either independent or conditionally dependent on the diagnostic group or specific symptoms. More research is needed to investigate the specific effect of ILF by using a waiting list, or better, an active control arm with an alternative treatment.

## Data Availability

The datasets presented in this article are not readily available because there was no consent given by the subjects to share those data publically or by request. Requests to access the datasets should be directed to kontakt@thomas-theis.de.
