# Supplementary material for: ILF-neurofeedback in clinical practice: examining symptom change and performance metrics across diagnostic groups
Source: Front Hum Neurosci. 2025 Jul 30;19:1601187. doi: 10.3389/fnhum.2025.1601187 (PMC12343661; doi:10.3389/fnhum.2025.1601187)
Supplement: Supplementary file 1 [file Table_1.docx]

Appendix B

***Symptom Frequencies***

| category | item | F3 | F4 | F8 | F9 |
| --- | --- | --- | --- | --- | --- |
| Sleep | Bruxism (teeth grinding) | 11 | 8 | 5 | 13 |
|  | Difficulty maintaining sleep | 16 | 17 | 4 | 21 |
|  | Night sweats | 3 | 9 | 5 | 7 |
|  | Restless leg | 2 | 3 | 0 | 3 |
|  | Night terrors | 2 | 0 | 0 | 1 |
|  | Difficulty waking | 9 | 13 | 6 | 41 |
|  | Snoring | 0 | 4 | 0 | 3 |
|  | Nocturnal enuresis (bed wetting) | 0 | 0 | 5 | 4 |
|  | Difficulty falling asleep | 11 | 19 | 10 | 35 |
|  | Dysregulated sleep cycle | 3 | 3 | 1 | 8 |
|  | Nightmares or vivid dreams | 7 | 11 | 1 | 16 |
|  | Restless sleep | 4 | 14 | 3 | 12 |
|  | Narcolepsy | 1 | 2 | 2 | 1 |
|  | Sleep - number of hours | 1 | 1 | 1 | 2 |
|  | Sleep quality overall | 3 | 3 | 0 | 6 |
|  | Periodic leg movements | 2 | 1 | 0 | 3 |
|  | Sleep apnea | 0 | 2 | 0 | 1 |
|  | Talking during sleep | 0 | 1 | 0 | 1 |
|  | Sleep walking | 0 | 0 | 2 | 0 |
| Attention & Learning | Difficulty completing tasks | 10 | 12 | 25 | 65 |
|  | Difficulty organizing personal time or space | 4 | 6 | 21 | 56 |
|  | Difficulty remembering names | 3 | 2 | 4 | 9 |
|  | Difficulty shifting tasks | 2 | 2 | 8 | 24 |
|  | Difficulty thinking clearly | 8 | 11 | 6 | 21 |
|  | Distractibility | 17 | 21 | 38 | 111 |
|  | Inattention | 3 | 2 | 24 | 69 |
|  | Lacking common sense | 0 | 0 | 6 | 10 |
|  | Not listening | 3 | 3 | 17 | 45 |
|  | Poor concentration | 10 | 21 | 29 | 81 |
|  | Poor short-term memory | 6 | 22 | 17 | 55 |
|  | Poor sustained attention | 8 | 12 | 26 | 92 |
|  | Unmotivated | 15 | 22 | 15 | 61 |
|  | Messy handwriting | 1 | 1 | 10 | 22 |
|  | Difficulty understanding conversations | 3 | 2 | 5 | 14 |
|  | Poor math | 1 | 2 | 10 | 14 |
|  | Slow thinking | 4 | 10 | 11 | 30 |
|  | Difficulty shifting attention | 1 | 4 | 12 | 26 |
|  | Difficulty making decisions | 12 | 17 | 8 | 37 |
|  | Reading difficulty | 3 | 4 | 15 | 20 |
|  | Lack of alertness | 7 | 4 | 2 | 19 |
|  | Poor verbal expression | 0 | 1 | 9 | 13 |
|  | Poor word finding | 1 | 8 | 5 | 7 |
|  | Poor vocabulary | 0 | 0 | 7 | 10 |
|  | Poor drawing ability | 0 | 0 | 8 | 12 |
|  | Reading difficulty | 0 | 0 | 0 | 1 |
|  | Motion sickness | 4 | 4 | 1 | 4 |
| Perception/ Sensory | Tinnitus (ringing in the ears) | 8 | 4 | 0 | 0 |
|  | Auditory hypersensitivity | 6 | 17 | 14 | 31 |
|  | Somatosensory deficits | 2 | 4 | 3 | 1 |
|  | Poor body awareness | 4 | 6 | 8 | 14 |
|  | Chemical sensitivities | 1 | 2 | 3 | 4 |
|  | Visual deficits | 2 | 3 | 2 | 0 |
|  | Visual hypersensitivity | 1 | 7 | 2 | 9 |
|  | Tactile hypersensitivity | 3 | 4 | 7 | 14 |
|  | Vertigo | 6 | 7 | 0 | 7 |
| Behavior | Excessive talking | 4 | 2 | 8 | 30 |
|  | Impulsivity | 9 | 4 | 18 | 55 |
|  | Nail biting | 2 | 2 | 7 | 28 |
|  | Poor eye contact | 0 | 0 | 8 | 16 |
|  | Undityness | 3 | 7 | 10 | 46 |
|  | Crying | 5 | 6 | 9 | 26 |
|  | Hyperactivity | 6 | 6 | 7 | 46 |
|  | Manipulative behavior | 1 | 1 | 3 | 13 |
|  | Oppositional or defiant behavior | 0 | 0 | 11 | 24 |
|  | Poor social or emotional reciprocity | 1 | 2 | 8 | 20 |
|  | Compulsive behaviors | 3 | 2 | 1 | 5 |
|  | Lack of appetite awareness | 2 | 3 | 1 | 3 |
|  | Rages | 5 | 4 | 16 | 41 |
|  | Class clown | 0 | 1 | 6 | 17 |
|  | Inflexibility | 5 | 7 | 5 | 22 |
|  | Lack of social interest | 9 | 14 | 4 | 14 |
|  | Aggressive behavior | 5 | 4 | 9 | 41 |
|  | Binging and purging | 2 | 3 | 4 | 20 |
|  | Poor speech articulation | 0 | 1 | 6 | 10 |
|  | Compulsive eating | 2 | 4 | 3 | 8 |
|  | Lack of sense of humor | 6 | 1 | 1 | 2 |
|  | Motor or vocal tics | 0 | 0 | 2 | 15 |
|  | Addictive behaviors | 5 | 3 | 2 | 3 |
|  | Autistic stimming | 0 | 0 | 1 | 4 |
|  | Stuttering | 4 | 2 | 3 | 6 |
|  | Anorexia | 1 | 3 | 0 | 0 |
| Emotion | Lack of social awareness | 1 | 0 | 2 | 18 |
|  | Depression | 21 | 21 | 1 | 18 |
|  | Mood swings | 12 | 9 | 6 | 18 |
|  | Irritability | 3 | 6 | 4 | 19 |
|  | Agitation | 5 | 4 | 2 | 13 |
|  | Anxiety | 12 | 24 | 8 | 35 |
|  | Dissociative episodes | 3 | 5 | 2 | 3 |
|  | Easily embarrassed | 2 | 2 | 2 | 6 |
|  | Emotional reactivity | 7 | 8 | 8 | 23 |
|  | Impatience | 4 | 9 | 11 | 45 |
|  | Lack of emotional awareness | 4 | 5 | 4 | 10 |
|  | Obsessive negative thoughts | 8 | 5 | 2 | 4 |
|  | Low self-esteem | 9 | 13 | 16 | 25 |
|  | Difficult to soothe | 4 | 8 | 6 | 24 |
|  | Lack of pleasure | 9 | 11 | 3 | 15 |
|  | Obsessive worries | 7 | 8 | 4 | 8 |
|  | Panic attacks | 4 | 7 | 3 | 2 |
|  | Suicidal thoughts | 4 | 3 | 2 | 0 |
|  | Fears | 6 | 9 | 4 | 15 |
|  | Anger | 0 | 2 | 6 | 12 |
|  | Flashbacks of trauma | 4 | 13 | 2 | 4 |
|  | Feelings of unreality | 0 | 2 | 0 | 1 |
|  | Paranoia | 4 | 1 | 0 | 2 |
|  | Mania | 0 | 1 | 0 | 0 |
| Physical | Irritable bowel | 6 | 9 | 1 | 6 |
|  | Poor gross motor coordination | 1 | 0 | 3 | 3 |
|  | Rigidity of Movements | 2 | 0 | 1 | 0 |
|  | Hot flashes | 1 | 2 | 0 | 2 |
|  | Seizures | 4 | 6 | 8 | 16 |
|  | Poor fine motor coordination | 1 | 1 | 10 | 8 |
|  | Tremor | 1 | 3 | 0 | 1 |
|  | Chronic constipation | 7 | 5 | 7 | 11 |
|  | PMS symptoms | 2 | 2 | 4 | 6 |
|  | Stress incontinence | 0 | 0 | 2 | 2 |
|  | Sweating | 2 | 2 | 1 | 4 |
|  | Low muscle tone | 0 | 0 | 2 | 2 |
|  | Poor balance | 1 | 3 | 1 | 1 |
|  | Allergies | 0 | 3 | 3 | 4 |
|  | Sugar craving and reactivity | 3 | 4 | 7 | 14 |
|  | Spasticity | 0 | 3 | 1 | 4 |
|  | Muscle tension | 9 | 13 | 0 | 13 |
|  | Asthma | 0 | 3 | 1 | 2 |
|  | Fatigue | 6 | 14 | 2 | 11 |
|  | High blood pressure | 1 | 0 | 0 | 1 |
|  | Urge incontinence | 1 | 1 | 3 | 2 |
|  | Reflux | 1 | 0 | 0 | 1 |
|  | Muscle weakness | 3 | 4 | 0 | 1 |
|  | Heart palpitations | 0 | 6 | 0 | 2 |
|  | Tachycardia (racing heart) | 1 | 2 | 1 | 0 |
|  | Effort fatigue | 0 | 0 | 0 | 1 |
|  | Immune deficiency | 1 | 1 | 2 | 0 |
|  | Difficulty working | 0 | 0 | 0 | 1 |
| Pain | Joint pain | 2 | 4 | 0 | 1 |
|  | Migraine headaches | 4 | 3 | 0 | 3 |
|  | Muscle tension headaches | 2 | 2 | 0 | 5 |
|  | Sinus headaches | 2 | 2 | 1 | 3 |
|  | Stomach aches | 0 | 2 | 0 | 4 |
|  | Chronic aching pain | 3 | 5 | 0 | 2 |
|  | Chronic nerve pain | 0 | 5 | 0 | 1 |
|  | Abdominal pain | 3 | 1 | 3 | 5 |
|  | Jaw pain | 0 | 1 | 0 | 1 |
|  | Fibromyalgia pain | 3 | 0 | 0 | 0 |
|  | Muscle pain | 1 | 2 | 0 | 2 |
|  | Ischias | 1 | 0 | 0 | 0 |
|  | Sciatica | 1 | 0 | 0 | 0 |
