# Supplementary material for: ILF-neurofeedback in clinical practice: examining symptom change and performance metrics across diagnostic groups
Source: Front Hum Neurosci. 2025 Jul 30;19:1601187. doi: 10.3389/fnhum.2025.1601187 (PMC12343661; doi:10.3389/fnhum.2025.1601187)
Supplement: Supplementary file 2 [file Table_2.docx]

Appendix C

contributions (c) bootstrap t-values (t) by Dimension and Items; values greyed out, for which t-values are −2 ≤ x ≤ 2.

| item | Dim1 | | Dim2 | | Dim3 | |
| --- | --- | --- | --- | --- | --- | --- |
|  | c | t | c | t | c | t |
| Oppositional or defiant behavior | -2.055 | -30.555 | 0.008 | 4.056 | 0.195 | 0.474 |
| Motor or vocal tics | -0.828 | -25.486 | 0.000 | 1.128 | -2.307 | -2.164 |
| Poor eye contact | -1.434 | -24.719 | 0.006 | 3.430 | 0.331 | 0.553 |
| Poor drawing ability | -1.268 | -22.323 | 0.007 | 3.725 | 1.432 | 1.211 |
| Poor vocabulary | -1.089 | -19.790 | 0.007 | 3.358 | 1.471 | 1.137 |
| Lacking common sense | -0.992 | -18.677 | 0.005 | 2.922 | 0.714 | 0.793 |
| Nocturnal enuresis (bed wetting) | -0.651 | -14.109 | 0.007 | 3.160 | 3.438 | 1.680 |
| Inattention | -3.758 | -11.428 | 0.119 | 1.222 | -0.180 | -0.636 |
| Autistic stimming | -0.264 | -8.045 | 0.000 | 0.896 | -0.167 | -0.373 |
| Not listening | -1.928 | -6.456 | 0.057 | 0.597 | 0.000 | -0.038 |
| Messy handwriting | -1.300 | -6.278 | 0.022 | 0.458 | 0.188 | 0.478 |
| Poor sustained attention | -1.959 | -6.168 | 0.000 | -0.070 | -1.289 | -1.987 |
| Class clown | -1.003 | -6.036 | -0.031 | -0.783 | -0.032 | -0.188 |
| Poor verbal expression | -1.100 | -5.985 | -0.022 | -0.626 | 1.841 | 1.310 |
| Stress incontinence | -0.276 | -5.963 | 0.002 | 1.698 | 0.972 | 0.819 |
| Difficulty organizing personal time or space | -1.895 | -5.877 | 0.001 | 0.014 | 0.000 | -0.014 |
| Low muscle tone | -0.276 | -5.316 | 0.002 | 1.592 | 0.972 | 0.747 |
| Distractibility | -1.093 | -5.020 | 0.078 | 0.644 | -0.066 | -0.703 |
| Lack of social awareness | -0.689 | -4.992 | 0.123 | 1.094 | -3.096 | -2.746 |
| Impulsivity | -1.139 | -4.406 | 1.170 | 2.264 | -0.197 | -0.645 |
| Poor speech articulation | -0.701 | -4.406 | -0.043 | -0.731 | 0.742 | 0.854 |
| Rages | -1.129 | -4.338 | 0.223 | 1.016 | 0.019 | 0.193 |
| Difficulty shifting attention | -0.988 | -4.106 | -0.141 | -0.946 | 0.325 | 0.653 |
| Nail biting | -0.874 | -4.070 | 0.031 | 0.415 | -0.881 | -1.189 |
| Poor social or emotional reciprocity | -0.827 | -3.818 | -0.005 | -0.166 | 0.017 | 0.111 |
| Difficulty shifting tasks | -0.788 | -3.631 | 0.037 | 0.415 | -0.081 | -0.359 |
| Poor fine motor coordination | -0.700 | -3.491 | 0.039 | 0.453 | 6.491 | 2.588 |
| Difficulty completing tasks | -0.785 | -3.458 | 0.069 | 0.487 | 0.049 | 0.382 |
| Untidyness | -0.808 | -3.391 | -0.132 | -0.750 | -1.989 | -1.995 |
| Poor math | -0.735 | -3.259 | -0.003 | -0.130 | 2.232 | 1.506 |
| Anger | -0.556 | -3.250 | -0.199 | -1.336 | 0.301 | 0.598 |
| Poor concentration | -0.598 | -3.197 | -0.377 | -1.310 | 0.000 | -0.034 |
| Aggressive behavior | -0.695 | -3.169 | 0.206 | 0.939 | -1.770 | -1.866 |
| Excessive talking | -0.671 | -3.159 | 0.401 | 1.294 | -0.641 | -1.077 |
| Reading difficulty | -0.617 | -2.694 | 0.013 | 0.222 | 3.779 | 2.179 |
| Sleepwalking | -0.202 | -2.542 | 0.005 | 2.542 | 5.243 | 2.542 |
| Hyperactivity | -0.412 | -2.499 | 0.106 | 0.610 | -4.030 | -3.162 |
| Impatience | -0.484 | -2.450 | -0.193 | -0.947 | -1.114 | -1.550 |
| Manipulative behavior | -0.362 | -2.414 | 0.015 | 0.282 | -0.536 | -0.980 |
| Binging and purging | -0.250 | -1.727 | -0.001 | -0.032 | -1.036 | -1.347 |
| Poor gross motor coordination | -0.145 | -1.247 | 0.401 | 1.068 | 1.335 | 1.033 |
| Crying | -0.108 | -1.096 | 0.033 | 0.344 | -0.003 | -0.053 |
| Difficulty understanding conversations | -0.124 | -1.091 | 0.269 | 0.774 | -0.001 | -0.082 |
| Slow thinking | -0.083 | -0.928 | -0.413 | -1.214 | 0.015 | 0.151 |
| Sugar craving and reactivity | -0.060 | -0.795 | 0.005 | 0.140 | 0.424 | 0.766 |
| Tactile hypersensitivity | -0.060 | -0.707 | 0.005 | 0.097 | 0.424 | 0.797 |
| Poor short-term memory | -0.029 | -0.630 | -2.284 | -3.053 | -0.089 | -0.414 |
| Urge incontinence | -0.010 | -0.310 | 0.041 | 0.271 | 2.129 | 1.451 |
| Difficulty remembering names | -0.008 | -0.291 | 0.346 | 0.772 | 0.111 | 0.409 |
| Seizures | -0.005 | -0.225 | -0.003 | -0.020 | 0.528 | 0.864 |
| PMS symptoms | -0.007 | -0.194 | 0.067 | 0.339 | 0.738 | 0.962 |
| Irritability | -0.003 | -0.163 | -0.134 | -0.565 | -0.562 | -1.094 |
| Difficult to soothe | -0.003 | -0.150 | -0.177 | -0.629 | -0.331 | -0.814 |
| Allergies | -0.004 | -0.136 | -1.072 | -1.834 | 0.802 | 0.938 |
| Chemical sensitivities | -0.002 | -0.077 | -0.037 | -0.255 | 0.763 | 0.867 |
| Poor body awareness | 0.000 | -0.050 | -0.004 | -0.091 | 0.946 | 1.194 |
| Abdominal pain | 0.005 | 0.184 | 1.074 | 1.327 | 0.389 | 0.707 |
| Inflexibility | 0.004 | 0.203 | 0.000 | 0.064 | -0.442 | -0.879 |
| Stomach aches | 0.009 | 0.222 | -0.869 | -1.557 | -0.803 | -2.182 |
| Easily embarrassed | 0.008 | 0.262 | 0.063 | 0.306 | 0.000 | 0.001 |
| Sleep - number of hours | 0.027 | 0.373 | 0.038 | 0.174 | 0.077 | 0.286 |
| Spasticity | 0.025 | 0.383 | -1.422 | -2.001 | -0.021 | -0.139 |
| Immune deficiency | 0.054 | 0.426 | 0.060 | 0.238 | 3.076 | 1.975 |
| Emotional reactivity | 0.018 | 0.431 | 0.083 | 0.438 | 0.004 | 0.052 |
| Compulsive eating | 0.025 | 0.449 | -0.109 | -0.383 | 0.026 | 0.220 |
| Jaw pain | 0.053 | 0.484 | -0.650 | -1.055 | -0.108 | -0.691 |
| High blood pressure | 0.065 | 0.524 | 1.167 | 1.085 | -0.142 | -0.893 |
| Talking during sleep | 0.053 | 0.535 | -0.650 | -1.136 | -0.108 | -0.704 |
| Reflux | 0.065 | 0.575 | 1.167 | 1.126 | -0.142 | -0.832 |
| Auditory hypersensitivity | 0.036 | 0.616 | -1.338 | -1.822 | 0.624 | 1.103 |
| Difficulty waking | 0.038 | 0.618 | -0.009 | -0.129 | -2.506 | -2.761 |
| Low self-esteem | 0.049 | 0.658 | -0.001 | -0.028 | 2.756 | 2.250 |
| Narcolepsy | 0.104 | 0.753 | -0.071 | -0.315 | 1.544 | 1.406 |
| Stuttering | 0.086 | 0.774 | 1.055 | 1.273 | 0.215 | 0.576 |
| Lack of alertness | 0.073 | 0.794 | 1.209 | 1.491 | -1.719 | -2.327 |
| Asthma | 0.124 | 0.821 | -1.894 | -2.094 | 0.124 | 0.430 |
| Sweating | 0.123 | 0.845 | 0.075 | 0.330 | -0.016 | -0.170 |
| Lack of emotional awareness | 0.109 | 0.854 | 0.016 | 0.103 | 0.084 | 0.327 |
| Agitation | 0.111 | 0.899 | 0.419 | 0.815 | -0.607 | -1.276 |
| Disregulated sleep cycle | 0.117 | 0.900 | 0.097 | 0.381 | -0.464 | -1.115 |
| Rigidity | 0.224 | 0.903 | 3.218 | 1.907 | 1.103 | 1.215 |
| Muscle tension headaches | 0.150 | 0.918 | 0.066 | 0.331 | -0.767 | -2.221 |
| Poor word finding | 0.151 | 0.962 | -2.569 | -2.348 | 1.248 | 1.358 |
| Unmotivated | 0.069 | 0.984 | -0.019 | -0.247 | -0.736 | -1.430 |
| Periodic leg movements | 0.182 | 1.020 | 0.611 | 0.728 | -0.390 | -1.513 |
| Chronic constipation | 0.151 | 1.064 | 0.950 | 1.175 | 1.166 | 1.411 |
| Visual hypersensitivity | 0.168 | 1.089 | -2.128 | -2.046 | -0.073 | -0.444 |
| Compulsive behaviors | 0.182 | 1.109 | 0.502 | 0.716 | -0.075 | -0.500 |
| Feelings of unreality | 0.244 | 1.122 | -1.733 | -1.850 | -0.029 | -0.429 |
| Sinus headaches | 0.199 | 1.124 | 0.085 | 0.299 | 0.008 | 0.111 |
| Sleep apnea | 0.244 | 1.166 | -1.733 | -1.893 | -0.029 | -0.377 |
| Night terrors | 0.288 | 1.231 | 3.113 | 1.902 | -0.063 | -0.608 |
| Hot flashes | 0.296 | 1.247 | -0.113 | -0.349 | -0.137 | -0.885 |
| Muscle pain | 0.296 | 1.296 | -0.113 | -0.312 | -0.137 | -0.838 |
| Snoring | 0.323 | 1.381 | -2.972 | -2.731 | -0.213 | -1.054 |
| Tachycardia (racing heart) | 0.432 | 1.387 | -0.123 | -0.237 | 1.059 | 1.360 |
| Lack of appetite awareness | 0.357 | 1.400 | -0.011 | -0.127 | 0.020 | 0.234 |
| Sleep quality overall | 0.352 | 1.472 | 0.112 | 0.378 | -0.746 | -2.138 |
| Poor balance | 0.448 | 1.581 | -0.565 | -0.679 | 0.424 | 0.885 |
| Somatosensory deficits | 0.442 | 1.615 | -0.183 | -0.445 | 2.603 | 1.987 |
| Difficulty thinking clearly | 0.371 | 1.788 | 0.000 | -0.015 | -0.018 | -0.211 |
| Night sweats | 0.496 | 1.794 | -1.227 | -1.372 | 1.255 | 1.469 |
| Fears | 0.428 | 1.805 | -0.024 | -0.205 | -0.024 | -0.204 |
| Restless leg | 0.557 | 1.885 | -0.017 | -0.150 | -0.177 | -1.044 |
| Visual deficits | 0.628 | 1.902 | -0.010 | -0.120 | 2.226 | 2.125 |
| Difficulty making decisions | 0.430 | 2.044 | -0.006 | -0.130 | -0.553 | -1.170 |
| Difficulty falling asleep | 0.466 | 2.080 | -0.255 | -0.741 | -0.037 | -0.315 |
| Dissociative episodes | 0.742 | 2.107 | -0.079 | -0.288 | 0.475 | 0.992 |
| Addictive behaviors | 0.778 | 2.209 | 1.436 | 1.207 | 0.424 | 0.990 |
| Motion sickness | 0.814 | 2.335 | 0.197 | 0.395 | 0.002 | 0.077 |
| Tremor | 0.766 | 2.475 | -0.715 | -0.755 | 0.000 | -0.079 |
| Paranoia | 0.817 | 2.521 | 3.533 | 1.741 | -0.069 | -0.746 |
| Mood swings | 0.791 | 2.569 | 1.556 | 1.570 | 0.024 | 0.222 |
| Obsessive worries | 1.042 | 2.732 | 0.117 | 0.367 | 0.445 | 0.963 |
| Heart palpitations | 0.963 | 2.806 | -5.846 | -4.455 | -0.008 | -0.266 |
| Lack of sense of humor | 1.031 | 2.809 | 6.529 | 2.910 | 0.126 | 0.633 |
| Bruxism (teeth grinding) | 1.046 | 2.816 | 1.701 | 1.482 | 0.145 | 0.518 |
| Migraine headaches | 1.055 | 2.835 | 0.725 | 0.760 | -0.098 | -0.888 |
| Suicidal thoughts | 1.163 | 2.917 | 0.869 | 0.825 | 1.911 | 2.122 |
| Nightmares or vivid dreams | 1.095 | 2.966 | -0.093 | -0.410 | -1.011 | -2.197 |
| Lack of pleasure | 1.253 | 3.045 | 0.048 | 0.264 | -0.093 | -0.458 |
| Panic attacks | 1.339 | 3.050 | -0.184 | -0.369 | 1.676 | 1.910 |
| Chronic nerve pain | 1.002 | 3.175 | -5.412 | -4.779 | 0.008 | 0.228 |
| Restless sleep | 1.193 | 3.199 | -2.852 | -2.206 | 0.000 | 0.014 |
| Vertigo | 1.614 | 3.548 | 0.071 | 0.310 | -0.350 | -1.682 |
| Obsessive negative thoughts | 1.736 | 3.705 | 2.312 | 1.448 | 0.291 | 0.922 |
| Lack of social interest | 1.715 | 3.734 | -0.099 | -0.388 | 0.019 | 0.259 |
| Anxiety | 1.210 | 3.755 | -0.946 | -1.497 | -0.197 | -0.720 |
| Chronic aching pain | 1.561 | 3.795 | -0.124 | -0.330 | -0.001 | -0.155 |
| Joint pain | 1.335 | 4.004 | -0.322 | -0.503 | 0.009 | 0.343 |
| Fatigue | 1.910 | 4.064 | -1.357 | -1.367 | -0.017 | -0.185 |
| Irritable bowel | 2.001 | 4.083 | -0.049 | -0.239 | 0.000 | -0.030 |
| Fibromyalgia pain | 0.970 | 4.453 | 7.010 | 4.453 | 0.030 | 4.453 |
| Muscle weakness | 1.644 | 4.608 | 0.000 | 0.050 | 0.015 | 0.375 |
| Muscle tension | 2.489 | 4.652 | -0.033 | -0.240 | -0.752 | -2.456 |
| Flashbacks of trauma | 2.593 | 4.658 | -3.218 | -1.927 | 0.476 | 1.350 |
| Difficulty maintaining sleep | 2.698 | 4.847 | 0.478 | 0.936 | -0.095 | -0.515 |
| Depression | 5.954 | 8.138 | 1.103 | 1.456 | -0.278 | -1.356 |
| Anorexia | 1.171 | 8.311 | -0.893 | -0.747 | 0.093 | 6.126 |
| Tinnitus | 3.716 | 108.641 | 4.905 | 1.762 | 0.182 | 12.687 |
