# Supplementary material for: ILF-neurofeedback in clinical practice: examining symptom change and performance metrics across diagnostic groups
Source: Front Hum Neurosci. 2025 Jul 30;19:1601187. doi: 10.3389/fnhum.2025.1601187 (PMC12343661; doi:10.3389/fnhum.2025.1601187)
Supplement: Supplementary file 3 [file Data_Sheet_1.pdf]

Version 2024
With kind permission from Ute Bolduan and Thomas Theis

Symptom Tracking

First and last name: **Date:**

Date of Birth:

Email address:

Address:

If necessary, name and signature of parent or legal guardian

Dear patient,

You or your child are starting ILF neurofeedback treatment (also known as the Othmer method) in our practice. We are delighted that you have decided to do this and thank you for your trust. We require your co-operation to ensure a successful treatment. We would like to ask you to answer the following approx. 150 questions before the first treatment begins. This involves naming and assessing your problems and symptoms at the start of treatment. Over the next few weeks, you will be asked to re-evaluate a relevant selection of these symptoms (approx. 5 to 25). This process is known as symptom tracking. The assessment must always be carried out by the same person or persons (i.e. always by yourself, or e.g. by your parents/caretakers together, a parent alone or a parent and the child together).
This is an assessment of the current relevant problems or restrictions in your life, which we assess before the beginning of a treatment and monitor during the course of the treatment. This provides us with valuable information for making adjustments during the therapy phase.

Please complete the form and answer the questions as described below.

Rating	Description
0	I do not have a problem with or this issue does not apply to me
1 - 5	Mild to moderate impairment; the effect of the symptom is noticeable and increasingly pronounced
6 - 8	Moderately severe to clearly pronounced symptoms that significantly impair the ability to cope with everyday life
9	Massive impairment and restriction of the ability to act
10	Unbearable impairment, extreme pain

Evaluate each symptom with a number.

Pos	Symptom	Observation	Rating
1	SLEEP		0-10
1.1	**Nightmares or vivid dreams**	Do you dream a lot? Do your dreams often contain threatening or saddening content? Do you continue to carry your dreams into the day and feel burdened by them?	
1.2	**Hours of sleep**	Do you sleep too much or too little? How problematic is this?	
1.3	**Difficulty waking up**	Is it difficult to get out of bed in the morning? Do you feel tired although you have slept enough - or even too much?	
1.4	**Leg movement - periodic**	Also known as Restless Legs Syndrome. This symptom describes involuntary movements in the legs. These often occur more frequently at night or at rest.	
1.5	**Bruxism - teeth grinding**	Do you experience excessive muscle tension in the jaw? Has your dentist prescribed or advised you to wear a bite splint?	
1.6	**Difficulty sleeping through the night**	Do you wake up once or several times during the night? Do you find it difficult or impossible to fall asleep again?	
1.7	**Difficulty falling asleep**	Do you have problems getting to sleep? Do you suffer from wandering thoughts, do you ruminate and cannot get to sleep as a result?	
1.8	**Night terrors**	Pavor nocturnus, also known as ‘sleep terror’ / ‘night terror’, is a sleep disorder in which those affected are startled out of sleep at night with a loud cry. The next morning, there is usually no memory of the experience.	
1.9	**Bedwetting at night - enuresis nocturna**	Involuntary enuresis without a physical cause (in school-age children)	
1.10	**Nocturnal sweating**	Excessive or noticeable sweating (despite appropriate clothing and bed linen for the temperature)	
1.11	**Narcolepsy - overwhelming sleepiness**	Severe or excessive daytime sleepiness, attacks of falling asleep	
1.12	**Sleep apnoea**	Do you stop breathing during your sleep? Ask your partner about this if necessary.	
1.13	**Sleep walking**	This is a phenomenon that usually lasts a few minutes, in which the person sleeping leaves the bed without waking up, walks around and sometimes also carries out activities.	
1.14	**Snoring**	Snoring can impair restful sleep. Ask your partner about this if necessary.	
1.15	**Speaking while asleep**	Does the possibility of speaking in your sleep lead to impairment of yourself or others?	
1.16	**Irregular sleep cycles**	Do you often go to bed at different times? Do you sleep for different lengths of time? Does this have a problematic effect on your ability to cope with everyday life?	
1.17	**Restless legs**	Does the regular tensing or twitching of your arms or legs during sleep or wakefulness affect your sleep quality or performance during the day?	
1.18	**Restless sleep**	Do you have the feeling that you cannot reach deep sleep, are always on the verge of waking up and are not refreshed? Do you move around a lot in your sleep, are you restless so that it affects your performance the next day?	

Pos	Symptom	Observation	Rating
2	ATTENTIVENESS + LEARNING		0-10
2.1	**Distractibility**	Do you find it difficult to stay focussed on a task when distractions occur?	
2.2	**Limited range of terms**	Do you have the feeling that you/your child often lack the words to express or describe something?	
2.3	**Slow thinking**	Is the speed of your thought process slower than it should be? Are you unable to finish your thought? Do you find it difficult to follow other people's thoughts?	
2.4	**Difficulty reading**	Does reading pose a problem, e.g. when reading aloud or analysing content?	
2.5	**Lack of alertness**	Do you feel it is difficult to reach an optimal level of alertness and maintain it as appropriate?	
2.6	**Lack of common sense**	Are you prone to irrational, self-harming behaviour? Do you have difficulties understanding social norms and rules?	
2.7	**Procrastination**	Do you often procrastinate? Do you put off important tasks? Do you distract yourself with unimportant tasks instead of completing the tasks that are important to you?	
2.8	**Poor math skills**	Do you have poor grades in maths or are math problems difficult for you to solve?	
2.9	**Poor focused attention**	Do you find it difficult to maintain concentration over a longer period of time?	
2.10	**Poor concentration**	Do you find it difficult to stay focused on a task?	
2.11	**Poor word-finding**	Is word finding a problem for you? Is finding the right word often out of reach for you?	
2.12	**Poor drawing skills**	Do you have problems drawing things?	
2.13	**Poor short-term memory**	Do you often or regularly forget what you have just done or wanted to do?	
2.14	**Poor listening skills**	Do you have a difficult time listening to a conversation or staying focused on a conversation for a longer period of time?	
2.15	**Verbal expression**	Do you have difficulty in everyday life to find the ‘right words’ to describe a situation or an issue?	
2.16	**Difficulty completing tasks**	Do you have difficulties completing a task you have started (e.g. work tasks, homework or assignments)?	
2.17	**Difficulty switching focus**	Do you have problems shifting your focus between different activities?	
2.18	**Difficulty making decisions**	Do you, for example, have problems deciding on one of two or more options? Are you afraid of making the wrong decision and therefore delay decisions for an unnecessarily long time?	
2.19	**Difficulty following instructions**	Do you have difficulties carrying out things you are asked to do (e.g. doing homework, tidying up, completing tasks in a professional context)?	
2.20	**Difficulty thinking clearly**	Do you find it difficult to think clearly and, thus, for example, make decisions?	
2.21	**Difficulty remembering names**	Are you unable to remember the names of people you should know?	

Pos	Symptom	Observation	Rating
**2**	**Continuation ATTENTIVENESS + LEARNING**		0-10
2.22	**Difficulty in organising themselves spatiotemporally**	Do you often forget necessary documents? Do you have problems attending appointments on time? In the case of school children: is there unfavourable learning behaviour before assignments or problems completing homework regularly?	
2.23	**Difficulty understanding conversations**	Do you find it difficult to follow the content of conversations or to grasp the meaning of a conversation?	
2.24	**Difficulty shifting tasks**	Do you have problems switching between activities that require your attention?	
2.25	**Unmotivated**	Do you often lack the desire to do things that you actually want to do or should do? Do you suffer from a lack of motivation?	
2.26	**Messy handwriting**	Do you have difficulty writing clearly and legibly?	

**3**	**SENSORY/PERCEPTION**		0-10
3.1	**Auditory hypersensitivity**	Are you sensitive to loud sounds and noise? How stressful is this in your everyday life?	
3.2	**Chemical sensitivity**	Are you sensitive to chemicals, e.g. fabric softener, latex, deodorant?	
3.3	**Lack of body awareness**	Do you have difficulties recognising whether you are exhausted, hungry, thirsty or full? Do you realise whether you should exercise or take it easy? Do you often have bruises or are you more prone to accidents?	
3.4	**Motoric weakness**	Do you feel like you do not have enough strength in everyday situations?	
3.5	**Dizziness/Vertigo**	Do you have problems with dizziness, even after a medical examination, e.g. problems with the ear, the vestibular system, the eye or the cervical spine?	
3.6	**Somatosensory deficits**	Do you feel any discomfort? For example, are you particularly sensitive to heat and/or cold? Do you often feel unpleasant sensations in your internal organs, e.g. your digestive system? Do you have numb body parts or feel a tingling sensation? Do you have the impression of being clumsy?	
3.7	**Tactile hypersensitivity**	Are you particularly sensitive to stimuli for touch (e.g. labels in clothing, scratchy clothing, hard food, walking barefoot)?	
3.8	**Tinnitus**	Do you suffer from ringing in the ears that hinders you in your everyday life?	
3.9	**Visual deficits**	Do you have poor, blurred, hazy or double vision (despite glasses)? Do you get headaches after working at a screen?	
3.10	**Visual hypersensitivity**	Are you sensitive to brightness, e.g. strong lights, flashing light or strong sunlight?	

**4**	**BEHAVIOUR**		0-10
4.1	**Aggressive behaviour**	Do you lash out at small things or do you often have arguments with others? Does your aggressive behaviour get in the way of your everyday life?	
4.2	**Anorexia**	Are you underweight? Have you been diagnosed with anorexia? Do you feel too fat, even though others keep telling you that this is not true?	

Pos	Symptom	Observation	Rating
4	Continuation: BEHAVIOUR		0-10
4.3	**Attachment problems**	Do you tend to become so emotionally attached to a person that they feel constricted? Are you very afraid of losing your partner? Do you want to be close to your partner all the time?	
4.4	**Autistic stimming**	When overstimulated, do you tend to make repetitive movements or sounds to self-soothe? (e. g. rocking back and forth, flapping your hands, humming or counting too loudly)	
4.5	**Excessive talking**	Do you tend to talk excessively? Do you keep talking even when no one answers and the other person is no longer listening?	
4.6	**Hyperactivity**	Do you have difficulty sitting still, do you have to keep moving all the time?	
4.7	**Impulsivity**	Do you tend to do things without thinking first?	
4.8	**Class clown**	Are/were you a class clown? Is this a problem for you?	
4.9	**Lack of appetite awareness**	Do you have trouble realising that you are actually hungry? Do you often not give in to a feeling of hunger?	
4.10	**Lack of sense of humour**	Do you have difficulties matching the good mood of others in social situations? Do you have the feeling that you lack a sense of humour? Is that a problem for you?	
4.11	**Lack of social interest**	Do you avoid contact with other people (e.g. groups)? Do you prefer to be alone? Does the idea of socialising make you feel uncomfortable or anxious?	
4.12	**Poor eye contact**	Do you find it difficult to maintain or make eye contact when speaking to others?	
4.13	**Manipulative behaviour**	Are you often trying to influence other people to do what you want them to do? Is this a problem for you or the people around you?	
4.14	**Motor and vocal tics**	Do you have tics of any kind, e.g. Tourette's syndrome, eye blinking, throat clearing, involuntary head twitching, etc.?	
4.15	**Nail biting**	Do you tend to bite your nails when you are nervous or bored, for example?	
4.16	**Oppositional or defiant behaviour**	Do you do things that are the exact opposite of what is expected of you? Does this problematic behaviour have a negative impact on your family life and/or partnership?	
4.17	**Poor articulation skills**	Do you speak indistinctly or too quickly? Do you have difficulty expressing yourself?	
4.18	**Poor social or emotional reception**	Do you have the feeling that people often react negatively to you in social contexts? For example, are you rarely invited out?	
4.19	**Self-harming behaviour**	Are you harming yourself in any way, even though you know it is hurting you (e.g. by scratching, burning or generally living an unhealthy lifestyle)?	
4.20	**Stuttering**	Do you stutter? Is it limiting your social interactions?	
4.21	**Addictive behaviour**	Do you have addictions of any kind? (e.g. alcohol, drugs, nicotine, coffee, binge eating or other addictions)	
4.22	**Inflexibility**	Are you easily unsettled when things do not go exactly according to plan? Is this a problem in your everyday life?	

Pos	Symptom	Observation	Rating
**4**	**Continuation BEHAVIOUR**		0-10
4.23	**Untidiness**	Do you have difficulty keeping your surroundings (e.g. home, workplace, school bag) tidy, even though you would like to? Do others perceive you as untidy?	
4.24	**Crying**	Do you cry a lot and even on minor occasions? Are you easily sad or emotionally affected? Does this hinder you in your everyday life?	
4.25	**Rages**	Are you quick to lose your temper when you are upset? Do you tend to shout or insult others?	
4.26	**Compulsive eating**	Is there an urge to eat all the time? Do you think about food very often? Is this so dominant that it interferes with your everyday life and you want to change it?	
4.27	**Compulsive behaviours**	Do you feel the urge to think certain thoughts or do certain things repetitively, e.g. repetition or checking compulsions (compulsive washing, frequently checking whether the front door is locked or the hob is switched off)?	

**5**	**EMOTIONAL**		0-10
5.1	**Agitation**	Do you experience physical agitation during periods of high emotional stress (e.g. conflict)?	
5.2	**Anxiety**	Is anxiety a problem in your everyday life? Do you experience anxiety?	
5.3	**Fears**	Do you often feel anxious or worried that something unpleasant will happen? Does this hinder you in your everyday life?	
5.4	**Depression, depressive mood**	Do you feel empty, undriven, depressed or joyless inside? Do you have difficulty feeling pleasure and joy?	
5.5	**Derealisation**	Do you feel that what you are experiencing does not correspond to reality? Do you experience feelings of alienation that cause you anxiety?	
5.6	**Dissociative episodes**	Do you often experience episodes of absence, when you do not feel like yourself and are you difficult to engage with during these episodes? Do you lose track of time during such episodes?	
5.7	**Emotional reactivity**	Does your reaction in some situations or to comments from others seem inappropriately violent, e.g. with anger, annoyance, sadness?	
5.8	**Irritability**	Are you often or easily irritated, angry, resentful or grumpy?	
5.9	**Easily embarrassed**	Do you have a problem with blushing? Are you easily embarrassed by things?	
5.10	**Lack of emotional awareness**	Do you have difficulty assessing your own emotional state? Do you have a problem recognising how you are feeling?	
5.11	**Lack of pleasure**	Do you feel that you have too little joy in your life?	
5.12	**Lack of social awareness**	Do you, for example, have difficulties recognising what behaviour is appropriate in a group?	

Pos	Symptom	Observation	Rating
5	**Continuation EMOTIONAL**		0-10
5.13	**Mania**	Do you experience episodes of highs during which you tend to do unreasonable, inappropriate or dangerous things? Do you receive appropriate feedback from your entourage?	
5.14	**Flashbacks of trauma**	Do you experience flashbacks of traumatic experiences you have had?	
5.15	**Low self-esteem**	Do you have low self-esteem? Do you have difficulties appreciating yourself the way you are? Is low self-esteem impacting your everyday life?	
5.16	**Panic attacks**	Do you have panic attacks? Do you experience sudden panic in your everyday life, even though there is no relevant cause?	
5.17	**Difficult to soothe**	Is it difficult for you to calm down when you are upset or angry?	
5.18	**Mood swings**	Do you experience sudden mood changes like anger, sadness or indifference caused by minor events? Do you often experience mood swings during your day?	
5.19	**Suicidal thoughts**	Do you currently have thoughts of ending your life?	
5.20	**Impatience**	Do you lose your patience quickly, e.g. when something does not work straight away? Does this affect your everyday life?	
5.21	**Paranoia**	Do you have obsessive thoughts that you are followed by, e.g. feeling persecuted or afraid of being poisoned? Do you believe people speak ill of you?	
5.22	**Anger**	Do you get overly angry? Do others talk to you about the fact that you are quick to fly off the handle?	
5.23	**Obsessive negative thoughts**	Do you have daily recurring thoughts that revolve around negative content?	
5.24	**Obsessive worries**	Do you often worry about someone - your children, your partner, your parents or strangers? Do you worry about things that could happen? Are these worries omnipresent or do they hinder you in your everyday life?	

6	PHYSICAL		0-10
6.1	**Allergies**	Do you have any allergies that interfere with your daily life?	
6.2	**Asthma**	Do you have asthma?	
6.3	**Chronic constipation**	Do you have difficulty passing hard or irregular stools?	
6.4	**Encopresis - loss of bowel control**	Do you have problems with your bowel control?	
6.5	**Skin irritation**	Do you suffer from neurodermatitis or other skin problems?	
6.6	**Heart palpitations**	Are you aware of your heart beats? Do you feel it beating too fast at times? Are you worried about this?	
6.7	**Hot flashes**	Do you experience hot flushes, do you sweat excessively? Is this a problem for you in your everyday life?	
6.8	**High blood pressure**	Do you experience high blood pressure?	
6.9	**Immune deficiency**	Do you tend to get sick easily or often compared to others? Do the illnesses (e.g. colds) last longer than for others?	
6.10	**Urge incontinence**	Do you suffer from incontinence with a sudden urge to urinate?	

Pos	Symptom	Observation	Rating
6	Continuation PHYSICAL		0-10
6.11	Seizures	Do you have epilepsy?	
6.12	Fatigue/exhaustion	Are you often tired or exhausted?	
6.13	Muscle tension	Do you often experience tension in your muscles (typically in the neck, shoulders or spine)?	
6.14	Muscle weakness	Do you feel that you have much less strength than before? Does this affect your everyday life?	
6.15	Muscle twitches	Do you experience involuntary, i.e. uncontrollable and sudden muscle movements?	
6.16	Low muscle tone	Do you generally suffer from low muscle tone?	
6.17	PMS symptoms	Do you experience physical or emotional symptoms around your period?	
6.18	Reflux	Do you experience heartburn regularly?	
6.19	Irritable bowel syndrome	Does your digestive system react sensitively to external circumstances such as food, excitement, stress, etc.?	
6.20	Rigidity	Do you feel stiff and limited in your mobility?	
6.21	Poor fine motor coordination	Do you have problems with your fine motor skills, e.g. when sewing, fastening buttons, picking up small objects from the floor?	
6.22	Poor gross motor coordination	Do you stumble frequently? Do you have problems with controlling your body, throwing or catching things?	
6.23	Poor balance	Do you have problems with your balance? Do you often fall?	
6.24	Effort fatigue	Do you feel like you get tired quicker than would be appropriate for your age and physical condition?	
6.25	Difficulty working	Do your symptoms interfere with your ability to complete your work?	
6.26	Difficulty walking or moving	Do you have problems climbing stairs or walking on uneven grounds? Do you have particular difficulties performing large movements (e.g. lifting an arm or leg)?	
6.27	Sweating	Do you sweat excessively, even without physical exertion?	
6.28	Spasticity	Do you suffer from spastic paralysis?	
6.29	Stress incontinence	Do you experience incontinence when you experience body stress?	
6.30	Tachycardia (racing heart)	Do you feel that your heart regularly beats too fast (more than 100 beats per minute), even though there is no cause such as physical stress or excitement?	
6.31	Tremor - involuntary shaking	Do you experience involuntary shaking?	
6.32	Nausea	Do you often feel nauseous even when there is no reason for it?	
6.33	Clumsiness	Do you feel like ‘a bull in a china shop’ or do you often bump into people, knock things over or drop them?	
6.34	Sugar craving and reactivity	Do you crave sugar often or have addictive behaviour when consuming sugar (e.g. gobbling chocolate from a jar of Nutella)? Does high sugar consumption change your mood?	

Pos	Symptom	Observation	Rating
**7**	**PAIN**		0-10
7.1	**Abdominal pain**	Do you experience pain or discomfort in your abdomen?	
7.2	**Chronic nerve pain**	Have you been diagnosed with nerve-related pain (e.g. in the arms or legs, back, face)? Does this pain interfere with your everyday activities?	
7.3	**Chronic pain**	Do you experience chronic pain? Does it interfere with your daily activities?	
7.4	**Fibromyalgia pain**	Have you been diagnosed with fibromyalgia by your doctor? Do you suffer from constant or frequent pain due to the condition?	
7.5	**Joint pain**	Do you suffer often or constantly from pain in your joints?	
7.6	**Sciatica**	Do you suffer from pain in the lower back or legs?	
7.7	**Jaw pain**	Do you regularly experience pain in your jaw?	
7.8	**Stomach**	Do you have problems with frequent or regular stomach pain?	
7.9	**Migraines headaches**	Has your doctor diagnosed you with migraine-like headaches? How much does the pain affect your everyday life?	
7.10	**Muscle pain**	Do you often experience muscle pain?	
7.11	**Tension headaches**	Have you been diagnosed with tension headaches by your doctor? How much does the pain affect your everyday life?	
7.12	**Trigeminal neuralgia**	Has your doctor diagnosed you with trigeminal neuralgia? If yes, please rate the intensity of the pain.	
7.13	**Recurring headaches**	Do you experience recurring headaches? Is the frequency of headaches a particular problem for you?	

Continue on the next page!

Pos	Symptom	Observation	Rating
**8**	**POSITIVE SCALE**		0-10

This scale refers to competences. A **low number** means that the competence is **very well developed**. A **high number** means that the ability is **not or poorly developed** and there is a need for optimisation.

8.1	**Attention**	Is it easy for you to concentrate on one thing for a longer period of time?	
8.2	**Balance**	Do you feel balanced?	
8.3	**Gratitude**	Can you feel gratitude for your life or even for small things?	
8.4	**Energy level**	Do you feel you have a satisfactory level of strength and energy?	
8.5	**Focus**	Can you concentrate/focus on the most significant part of a situation?	
8.6	**Good quality of sleep**	Does the quality of your sleep match your needs?	
8.7	**Harmony**	Do you feel fulfilled in harmony?	
8.8	**Compassion**	Can you feel compassion for other people, e.g. when they are sad or in pain?	
8.9	**Optimism**	Are you an optimistic person?	
8.10	**Positive attitude**	Do you have an overall positive attitude?	
8.11	**Resilience**	Do you assert yourself? Do you feel you can overcome adverse circumstances in your life fairly easily?	
8.12	**Calmness**	Are you capable of regularly creating a feeling of calmness and tranquillity within yourself?	
8.13	**Self-confidence**	Are you a self-confident person?	
8.14	**Connectedness**	Do you feel connected to e.g. nature, your home, your family, friends?	
8.15	**Vitality**	Are you bursting with vitality? Do you do a lot?	
8.16	**Goal setting**	Do you feel that you are able to achieve your goals?	
8.17	**Happiness**	Are you happy with your life?	

Thank you for your cooperation!
